# Effect of Unsaturated Fatty Acids on Intra-Metabolites and Aroma Compounds of *Saccharomyces cerevisiae* in Wine Fermentation

**DOI:** 10.3390/foods10020277

**Published:** 2021-01-30

**Authors:** Peitong Liu, Violeta Ivanova-Petropulos, Changqing Duan, Guoliang Yan

**Affiliations:** 1 Centre for Viticulture and Enology, College of Food Science and Nutritional Engineering, China Agricultural University, Beijing 100083, China; pt_liu@cau.edu.cn (P.L.); chqduan@cau.edu.cn (C.D.); 2 Laboratory of Viticulture and Enology, Ministry of Agriculture and Rural Affairs, Beijing 100083, China; 3 Nutrition & Health Research Institute, COFCO Corporation, Beijing 102209, China; 4 Faculty of Agriculture, University “Goce Delčev”—Štip, Krste Misirkov 10-A, 2000 Štip, North Macedonia; violeta.ivanova@ugd.edu.mk

**Keywords:** unsaturated fatty acids, *Saccharomyces cerevisiae*, aroma compounds, red wine, intra-metabolites

## Abstract

The small changes in concentration of unsaturated fatty acids (UFAs) cause a significant influence on the aromatic component of wines. In this work, the effect of UFAs mixture (including linoleic, oleic, and α-linolenic acids) addition on intra-metabolites and aromatic compounds of two *Saccharomyces cerevisiae* strain EC1118 and BDX were investigated in red wine fermentation, respectively. The results showed that the pre-fermentative addition of UFAs significantly modified the physiological and energetic state of cells, and affected the levels of intra-metabolites in glycolysis pathway and TCA cycle, redox balance, ATP pool, fatty acids, and amino acids metabolism, which consequently altered the chemical and volatile composition of the wines. Different with the control wine, the wines produced by UFAs addition were characterized with higher amounts of glycerol, C6-alcohols and higher alcohols, and lower levels of acetic acid, medium-chain fatty acids, and acetate esters. Interestingly, the production of ethyl esters showed opposite profiles in different strains due to the distinct expression of *EEB1*, indicating that the effect of UFAs on ethyl esters syntheses is strain-specificity. Our results highlighted the effectiveness of modulating UFAs content in shaping aroma characteristics, and verified that fine adjusting the content of UFAs combined with inoculating proper yeast is a promising strategy to modulate the aromatic quality of wine, which probably provides an alternative approach to meet the expectations of wine consumers for diverse aromatic qualities.

## 1. Introduction

The fermentative aromas compounds (including esters, higher alcohols, and volatile fatty acids) are one of the principal attributes determining wine quality and influence the preferences of wine consumers [1]. Besides the strains of *Saccharomyces cerevisiae*, nutritional status of grape musts largely influences the aroma quality of the final wine products. The physiology and metabolism of wine yeasts can be affected significantly with even small changes in the must composition, and consequently alter the formation of fermentative aroma compounds [2]. Until now, much work has been carried out to investigate the effect of sugar (glucose and fructose), nitrogen (ammonium or amino acid), and carboxylic acids (malic and tartaric acid) on aroma compounds syntheses during wine fermentation [3,4,5], however, relatively little information is available on the function of unsaturated fatty acids (UFAs). UFAs is necessary for yeast under anaerobic conditions to maintain membrane integrity and adapt well to various environmental stresses during wine fermentation [6]. Our prior work indicated that UFAs can directly influence the synthesis of yeast-derived volatile compounds through regulating the expressions of related functional genes [7,8].

It should be pointed out that although there are several studies investigating the impact of UFA variation on aromas biosynthesis during wine fermentation, the reported results are always inconsistent. For example, Rollero et al. [9] found that adding oleic acid and ergosterol increased most of higher alcohols and acetate esters production, while Pinu et al. [10] found that increasing the content of linoleic acid improved the production of C6 compounds and higher alcohols, but decreased acetate esters and some ethyl ester. In the study of synergistic effect, pre-fermentative addition of Tween 80 (containing 70% of oleic acid and 30% of stearic acid and palmic acid) increases the higher alcohols, esters, and volatile fatty acids of Chardonnay wine [11], which are partially in agreement with the results of Duan et al. [12] in synthetic grape must MS300 with mixed addition of oleic acid, linoleic acid, and linolenic acid. In addition, the levels of major fermentative products (such as ethanol, glycerol, and acetic acid) are also varied. Obviously, these inconsistent results could be put down to the differences in amount and composition of UFAs added, fermentation conditions, grape medium, and wine yeast used. To rational modulate the levels of chemical and aromatic compounds by fine controlling UFAs, it is essential to elucidate their regulation mechanism. It is well-known that the syntheses of aroma volatiles are not only associated with functional gene expressions, but also the availability of precursors, the cellular redox potential, and the energy status of the cells [13]. Thus, the metabolism of yeast in response to UFAs variation needs to be characterized. In one recent study, Pinu, Villas-Boas, and Martin [14] investigated the effect of single fatty acid on the metabolism of wine yeast in white wine fermentation, and reported that the addition of different fatty acids affected the metabolism and growth of wine yeasts in a different way. Actually, UFAs in grape must contain a mixture of linoleic acid (C18:2, LA), oleic acid (C18:1, OA), and α-linolenic acid (C18:3, ALA). Their concentrations vary with grape cultivar [15] and fermentation procedures [16], and it is thus essential to investigate their synergistic effect on yeast overall metabolism during wine fermentation. To our knowledge, there are no relevant research reports yet.

In this work, the mixture of LA, OA, and ALA were pre-fermentative added in Cabernet Sauvignon juice. Considering that different wine yeast strains might show distinct metabolism profiles when response to the same nutritional condition and produce wines with diverse aroma compounds due to the yeast’s genetic variations, two wine yeast strains (EC1118 and BDX) were used in this study, respectively. The fermentation performance, extracellular volatile and non-volatile compounds, and intracellular metabolites were determined during the whole fermentation. The impacts of UFAs variation on intracellular metabolites and aromas syntheses was systemically investigated during red wine fermentation, to gain deep understanding of the aromatic function of UFAs in wine fermentation.

## 2. Materials and Methods

### 2.1. Chemicals and Reagents

All chromatographic reagents were of HPLC grade. n-Hexane, methanol, acetonitrile, and formic acid were bought from J.T. Baker (Phillipsburg, NJ, USA). Oleic acid, linoleic acid, and α-linolenic acid were purchased from Sigma-Aldrich (St. Louis, MO, USA). The standards (purity > 95%) used for the identification and quantification of glycerol, ethanol, glucose, organic acids, fatty acids (including dodecanoic acid, tetradecanoic acid, hexadecanoic acid, 9-hexadecenoic acid, octadecanoic acid, oleic acid, linoleic acid, α-linolenic acid, eicosanoic acid, and docosanoic acid), and volatile compounds (including 1-hexanol, (E)-3-hexen-1-ol, (Z)-3-hexen-1-ol, 1-propanol, isobutyl alcohol, isoamyl alcohol, 2,3-butanediol, 1-octanol, 1-octyl-3-ol, 1-decanol, benzyl alcohol, 2-phenylethanol, ethyl acetate, isoamyl acetate, hexyl acetate, phenethyl acetate, hexanoic acid, octanoic acid, decanoic acid, isobutyric acid, ethyl butanoate, ethyl hexanoate, ethyl octanoate, ethyl decanoate, ethyl dodecanoate, ethyl tetradecanoate, ethyl hexadecanoate, methyl octanoate, isoamyl hexanoate, propyl octanoate, citronellol, 3-(methylthio)-1-propanol), and β-damascenone) were also obtained from Sigma-Aldrich. Yeast extract, peptone, and analytical glucose were purchased from Sangon Biotech (Shanghai, China). Analytical reagent ethanol, tartaric acid, sodium hydroxide, hydrochloric acid, and trichloromethane were obtained from Sinopharm Chemical Reagent Beijing Co. Ltd. (Beijing, China).

### 2.2. Fermentation Medium and Yeast Strains

Ten kilograms of *Vitis vinifera* L. Cabernet Sauvignon, collected in the Changli wine region of China, were destemmed and crushed by hand, and the grape juices were drawn. After being pasteurized for 10 min at 72 °C, the grapes juice was used for wine fermentation. The initial sugar concentration was 241.3 g/L, and oleic acid (OA), linoleic acid (LA), and α-linolenic acid (ALA) in juice were 0.55 mg/L, 2.12 mg/L, and 0.81 mg/L, respectively. Two levels of UFAs were designed and initially added in pasteurized grape juice. The high-UFA concentration juice contains 240 mg/L LA, 70 mg/L OA, and 100 mg/L ALA. The low-UFA concentration juice contains 24 mg/L LA, 7 mg/L OA, and 10 mg/L ALA. The origin juice was used as a control. All initial concentrations of UFAs in juice were within the common range in fresh grape juice and must [12,17]. The commercial wine yeast *S. cerevisiae* strains EC1118 (E, Lallemand, Blagnac, France) and BDX (B, Lallemand, Blagnac, France) were used in this study.

### 2.3. Fermentation Conditions

*S. cerevisiae* was inoculated into 500 mL yeast extract peptone dextrose medium (5 g/L yeast extract, 20 g/L glucose and 10 g/L peptone), shaken (150 rpm), and incubated at 30 °C overnight. The yeast cells suspension was harvested and washed twice with sterile water, then added into the flask. The initial viable population was approximately 10^6^ CFU/mL. Nitrogen was sparged to eliminate oxygen from the medium before the inoculation. Triplicate fermentations were carried out in 500 mL flasks with 350 mL pasteurized juice at 25 °C without shaking. The flasks were equipped with fermentation locks to achieve anaerobic conditions and a puncture needle for sampling. The fermentation progress was monitored by sugar consumption. Dry cell weight (DCW) was calculated with DCW (g/L) = 0.3 × OD_600nm_ (optical density at 600 nm) according to our preliminary work [8]. A volume of 30 mL sample of culture medium was taken from fermenting juice in mid-exponential (about 20% sugar consumption), early-stationary (about 50% sugar consumption), late-stationary (about 80% sugar consumption) phase and in the end of fermentation. The samples were centrifuged at 13,800× *g* for 10 min to harvest yeast cells and supernatants. After that, cells were kept at −80 °C for RNA isolation and intracellular metabolites analysis, while supernatants were kept at −20 °C for the analysis of non-volatile and volatile compounds.

### 2.4. Analysis Methods

#### 2.4.1. Major Fermentation Products Analyses

Total sugar was detected according to the National Standard of the People’s Republic of China [18]. Glycerol, ethanol, citric acid, acetic acid, malic acid, and succinic acid in final wines were determined by high-performance liquid chromatography (HPLC, Agilent Technologies, Santa Clara, CA, USA). An HPX-87H Aminex ion-exchange column (300 mm × 7.8 mm, Bio-Rad Laboratories, Hercules, CA, USA) was used as described previously [8]. The calibration was previously performed with pure standard chemicals in deionized water. The calibration curves and *R*^2^ of glycerol, ethanol, citric acid, acetic acid, malic acid, and succinic acid used in this study are provided in Table S1.

#### 2.4.2. Fatty Acids Analyses

The intracellular fatty acids were analyzed according to the method of Duan et al. [12]. A mass of 5–10 mg dry weight yeast cells was put in sealed tubes and saponified at 100 °C for 30 min with 1 mL 5% NaOH (in 50% methanol/water). A volume of 2 mL HCl (6 M) was added into the cooled tubes. A volume of 0.5 mL mixture of hexane: Methyl-tert-butyl ether (1:1, *v/v*) was used to extract the free fatty acids. The analysis was performed on a 6890A gas chromatograph and a column HP-INNOWAX (60 m × 0.25 mm × 0.25 μm) equipped with a 5975C mass spectrum system (GC-MS, Agilent Technologies, Santa Clara, CA, USA). Pure standard chemicals in hexane were used to perform the calibration. Analyses were carried out in triplicate. The detailed quantitation information about quantitative standards, quantitative ion, calibration curves, and *R*^2^ for the quantification of volatile compounds is provided in Table S2.

#### 2.4.3. Volatile Compounds Analyses

Solid-phase micro-extraction and GC-MS was used to extract and analyze volatile compounds in wine, based on previously developed methods [8,19]. A SPME fiber (50/30 μm DVB/CAR/PDMS, Supelco, Bellefonte, PA, USA) was activated at 250 °C prior sample extraction. Then, 5 mL of wines were equilibrated at 40 °C for 30 min. After that, the SPME fiber was inserted into the headspace of the vial and extracted the volatile compounds at 40 °C for 30 min. Then, samples were analyzed by an Agilent 6890A and a column HP-INNOWAX (60 m × 0.25 mm × 0.25 μm) equipped with a 5975C MS system. A flow rate of 1.0 mL/min helium was used as the carrier gas. The injector temperature was 250 °C. Oven temperature was maintained at 50 °C for 1 min, and raised to 220 °C at a rate of 3 °C/min (held for 5 min). The temperature of ion source was 250 °C. Electron ionization (electron impact mode at 70 eV) MS data from *m/z* 30 to 350 was collected. Analyses were applied in triplicate. The qualification and quantification processes were based on our previous lab work [8]. A synthetic matrix was used as standard solutions, which was prepared in distilled water with 200 g/L glucose and 7 g/L tartaric acid. pH was adjusted to 3.3. The calibration curves of aroma standards were obtained by successive dilution of fifteen levels in the matrix. The detailed quantitation information about quantitative standards, quantitative ion, calibration curves, and *R*^2^ for the quantification of volatile compounds is provided in Table S3.

### 2.5. Metabolome Analysis

Samples for metabolome analysis were prepared according to previous literature [20]. A volume of 500 μL methanol and 10 μL ribitol (2 mg/mL in water, internal standard), were added to the yeast cells and shaken at 2000 rpm for 1 h. After the collection of the supernatant by centrifugation, 500 μL of water was added into the sediments and shaken (2000 rpm) for 1 h. The supernatant was collected and merged with the previous one. Then, a volume of 100 μL of supernatant was separated for the precise analysis of the intracellular amino acids and energy metabolites by UPLC-MS/MS (Thermo Scientific™ Ultimate 3000™ Q Extractive™, Waltham, MA, USA).

The UPLC-MS/MS system equipped with ACQUITY UPLC BEH Amide (1.7 μM100deY UPLC BEHcnal, Milford, MA, USA) column was used for analyses of intracellular amino acids. The separation and analysis of intracellular main pathway and energy metabolites was performed on an ACQUITY UPLC HSS T3 C18 column (1.7 μm, 2.1 × 100 mm, Waters, Milford, MA, USA). All the compounds were identified by comparison of the retention time and *m/z* values to library entries of purified standards or recurrent unknown entities. The calibration was performed with pure standards, and analyses were carried out in triplicate. All the detailed quantitation information about quantitative standards, quantitative ion, calibration curves, and *R*^2^ for the quantification of metabolites is provided in Tables S4 and S5.

### 2.6. Real-Time qPCR Assay

The relative expression level of *EHT1* and *EEB1* was measured by real-time PCR using TB Green^®^ Premix *Ex Taq*™ (Takara, Dalian, Liaoning, China) on an ABI 7300 Real-Time PCR System (Applied Biosystems, Foster City, CA, USA), with quantitative PCR primers (Table S6). The detailed descriptions of these genes are also listed in Table S6. *PDA1* and *ACT1* were taken as internal controls. The specificity of the primers was confirmed via melting curve analysis, and the relative gene expression levels were performed using 2^−∆∆Ct^ method [21]. For each sample, three independent extraction procedures and two technical replications of real-time PCR analysis were applied.

### 2.7. Statistical Analysis

Results were statistically treated by calculation of means, standard deviation, and relative standard deviation. The one-way analysis of variance (ANOVA) of multiple comparisons of mean values was applied to the results to test possible significant differences in metabolites among treatments, applying the Duncan test (*p* < 0.05), using the SPSS20.0 software (SPSS, Chicago, IL, USA). Principle component analysis (PCA) based on a correlation matrix was performed by SIMCA 14.1 (Umetrics, Malmö, Sweden) software. Data were normalized with mean-centered and divided by the standard deviation of each variable. Five principal components were extracted from forty-one compounds, and the first two were chosen for further analysis. The rest graphs were performed by Microsoft Office Excel 2016 (Microsoft Corporation, Redmond, WA, USA) and Origin 9.0 (OriginLab Corporation, Northampton, MA, USA).

## 3. Results

### 3.1. Effects of Pre-Fermentative Addition of UFAs Mixture on Yeast Growths, Fermentation Activity and Major Fermentation Products

**Figure 1:**
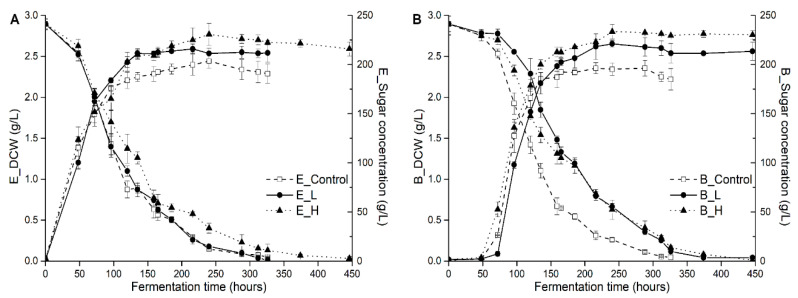
Cell growth and sugar consumption of EC1118 (**A**) and BDX (**B**) treated by different levels of UFAs during alcoholic fermentation (Control, L, and H represented the control, low-UFAs, and high-UFAs added juice).

The profiles of cell growth and sugar consumption (glucose and fructose), and some fermentation parameters (including fermentation duration time, maximum biomass, maximum growth rate, and fermentation rate) in different treatments are showed in Figure 1 and Table 1, respectively. In all wines, fermentation finished successfully to dryness (below 4 g/L residual sugar) within the monitored period of 326–446 h, and both yeast strains broadly followed similar growth patterns, while the fermentation kinetics was significantly different due to the UFAs addition. In fact, UFAs addition promoted yeast growth and population, but unexpectedly decreased fermentation rate (sugar consumptions rate, from 1.94 g/L/h in the control to 1.61 g/L/h in high-UFAs fermentation, on average) and prolonged fermentation duration time compared to the control, especially in BDX strain (326 h vs. 446 h in low-UFAs and high-UFAs added fermentations). The strain of BDX is more sensitive to UFAs addition than EC1118 strain because its fermentation duration time was extended by 120 h relative to EC1118 strain in low-UFAs added fermentation, which might explain the great variation of chemical and aromatic compounds in final wine as showed below. In general, the effect of UFAs addition on fermentation performances is strain-dependent, with a prolong effect of low addition on the fermentation duration of BDX, but not for EC1118.

The extracellular metabolites concentrations in wines at the end of fermentation are showed in Table 1, including glycerol, ethanol, citric acid, malic acid, succinic acid, and acetic acid. The pre-fermentative addition of UFAs influenced the final contents of glycerol, ethanol, and acetic acid in respective strain fermentation, but had a minor impact on succinic acid, citric acid, and malic acid productions. The contents of glycerol and ethanol reached the highest concentration in wines with high-UFAs addition (10.57 and 10.45 g/L, 14.57 and 14.72% *v/v* in EC1118 and BDX strains fermentation wines, respectively), while acetic acid contents were lowest in these wines regardless of the yeast strains (0.43 and 0.24 g/L, respectively), implying that UFAs has a special mechanism of influencing the syntheses of the major fermentative products. These results are inconsistent with the data of Pinu et al. [14], who reported that linoleic acid addition resulted in a promotion on acetic acid synthesis by *S. cerevisiae* EC1118. The inconsistent results could be due to the difference in added UFAs composition (single vs. synergistic effect), which needs to be further investigated.

### 3.2. Effects of Pre-Fermentative Addition of UFAs Mixture on Volatile Compounds Production

A total of thirty-five yeast-derived volatiles were determined in all wine in the end of alcoholic fermentation, including three C6-alcohols, nine higher alcohols, four fatty acids, fourteen esters (four acetate esters, seven ethyl esters, and three esters), two terpenes, one sulfide, and two aldehydes compounds (Table S7). Sixteen aroma compounds with odor activity values (OAVs) exceeding one are highlighted and presented in Table 2, and the total amount of five group volatiles (C6 alcohols, higher alcohols, acetate esters, medium fatty acid, and ethyl esters) are also showed. Unlike major fermentation products, the initial addition of UFAs significantly influenced the most aromas produced by two *S. cerevisiae* strains. Some of them follow similar patterns independent of fermented strains, while others showed strain-specific modes. Generally, the synthesis of C6-alcohols (1-hexanol, (E)-3-hexenyl-1-ol, (Z)-3-hexenyl-1-ol) and higher alcohols (isoamyl alcohol, isobutyl alcohol, 1-octyl-3-ol, 1-decanol, benzyl alcohol, and 2-phenylethanol) was improved, observing the highest content of these compounds in high-UFA wines regardless of the yeast strain, compared to the control, while the content of medium-chain fatty acids (hexanoic acid, octanoic acid, decanoic acid) and acetate esters (ethyl acetate, isoamyl acetate, hexyl acetate, phenethyl acetate) decreased. Surprisingly, a clear strain-specificity was found in the productions of ethyl esters, as it showed that their total contents was decreased by 30.0% and 22.8% in EC1118 strain fermentation, while they increased by 18.7% and 31.7% in the strain of BDX with the rise of UFAs level relative to the control wine. The affected ethyl esters are ethyl octanoate, ethyl decanoate, and ethyl dodecanoate, especially ethyl decanoate which, accounting for the total content of ethyl esters 52.9–62.8%, showed 23.3% and 19.2% decrement in low and high-UFAs wines fermented with EC1118 strain relative to the control wine, respectively, while the values were increased by 30.6% and 50.3% in BDX strain fermented wines, respectively (Table S7). Methyl octanoate showed similar trends (decreasing in EC1118 strain wine and increasing in BDX strain wine with the improvement of UFAs addition). These distinct profiles suggested that the effect of UFA on ethyl esters syntheses is strain-specificity. To reveal the mechanism at the molecular level, we determined the relative expression levels of *EHT1* and *EEB1* encoding acyl-CoA: ethanol O-acyltransferases, which are responsible for the biosynthesis of MCFA ethyl esters, at different stages of fermentation (including mid-exponential phase, the early-stationary phase, and the mid-stationary phase) by real-time PCR. The results shows that UFAs addition had no clear effect on *EHT1* expression, while the *EEB1* expression level was up-regulated by 1.2–1.4 fold in BDX strain while down-regulated by 1.8–3.3 fold in EC1118 strain compared to the control trials.

**Figure 2:**
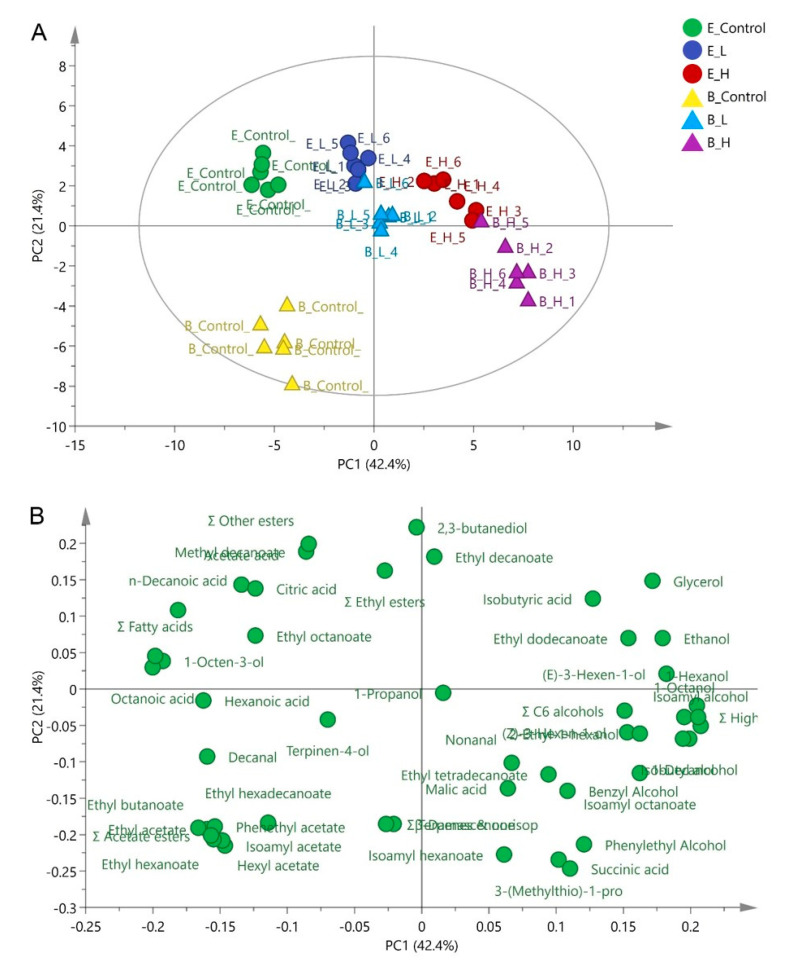
Principle components analysis using thirty-five volatile compounds and major fermentation products (including ethanol, glycerol, acetic acid, malic acid, citric acid, and succinic acid) in wines treated by different levels of UFAs ((**A**) Scatter plot; (**B**) loading plot). E_Control, E_L, and E_H represented the control, low-UFAs, and high-UFAs added wines fermented by EC1118 strain, respectively; B_Control, B_L, and B_H represented the control, low-UFAs, and high-UFAs added wines fermented by BDX strain, respectively.

Principal Component Analysis (PCA) (Figure 2) was applied using major fermentation products (Table 1) and all aromatic compounds listed in Table S7, to explore the distinct effect and identify the chemical and volatile compounds that discriminate these treatments. The first and second accounted for 42.4% (PC1) and 21.4% (PC2) of the total variation, respectively. The PC1 distinguished the high-UFAs added wines with no-UFAs addition wines. The wines without UFAs addition (E_control and B_control) were positioned on the negative part of PC1, separated by volatile fatty acids (octanoic acid, decanoic acid, and total fatty acids). The wines fermented with high-UFAs added juice (E_H and B_H) located on the positive side of PC1 and were separated by isoamyl alcohol and total higher alcohols. PC2 has the potential to discriminate the wine produced by different strains, mainly by 2,3-butanediol, ethyl decanoate, and isoamyl hexanoate. PCA analysis clearly indicated that high-UFAs addition wine and the control wines had the largest differences in chemical and aromatic composition. Comparative targeted intra-metabolites profiles (E_H vs. E-Control and B_H vs. B-Control) were therefore conducted between these two wines in the following study.

### 3.3. Effects of Pre-Fermentative Addition of UFAs Mixture on Intra-Metabolite Profile 

**Figure 3:**
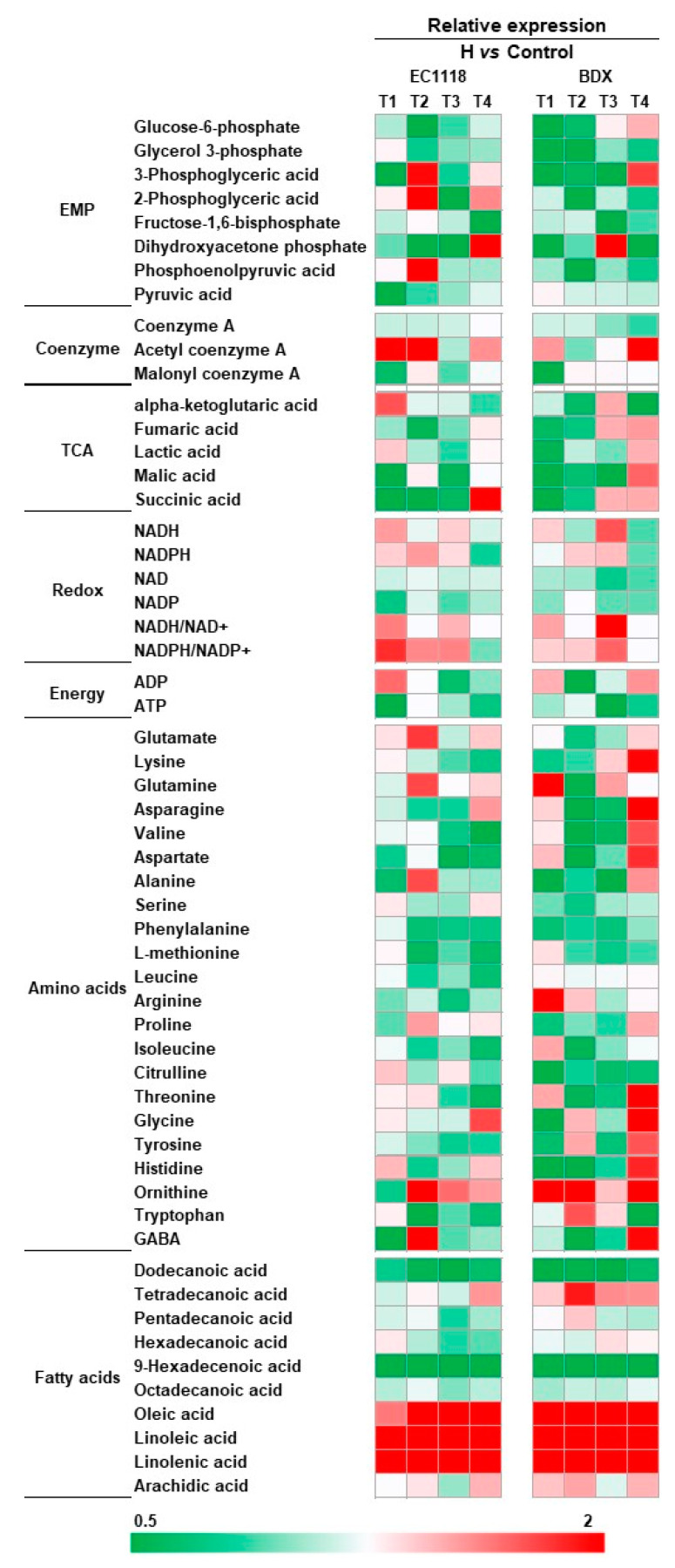
The comparison of intracellular metabolites produced by EC1118 and BDX strains in high-UFAs addition vs. the control wines fermentation. T1, T2, T3, and T4 represent the mid-exponential phase, the early-stationary phase, the mid-stationary phase, and the end of fermentation, respectively.

Samples for intracellular metabolite analysis were taken at four stages in fermentation, which corresponded to around 20%, 50%, 80% sugar consumption, and the end of the fermentation, respectively. A total of fifty-five intracellular metabolites were identified, including twenty-three glycolysis and TCA cycle intermediates, twenty-two amino acids, ten fatty acids, and energy pool, which are presented by heat map in Figure 3. UFA addition resulted in significant variation of metabolite levels in central carbon and nitrogen metabolisms, which are largely associated with the strain and fermentation stages. In general, the relative abundance of intermediates in glycolysis and TCA cycle, amino acids, ATP, and saturated fatty acid were decreased, especially before around 50% sugar consumption, while the levels of NADH, NADH/NAD^+^, NADPH, NADPH/NADP^+^, and unsaturated fatty acids were increased compared to the control wine samples. In the glycolysis pathway, the decreased intermediates included glucose-6-phosphate, 3-phosphoglyceric acid, glycerol 3-phosphate, fructose-1, 6-biophosphate, phosphoenolpyruvic acid, and pyruvic acid, which were profound in BDX strain. In comparison, the levels of TCA cycle intermediates are dependent on fermentation stage. In the early stage (corresponding to around 50% sugar consumption), the abundance of ketoglutaric acid, fumaric acid, malic acid, and succinic acid decreased in both strains, but were improved after cells entered the late stage (corresponding to around 80% sugar consumption), especially in the strain of BDX. Noticeably, we found that the ATP level in cells showed decrement in UFAs added fermentation, suggesting that energy is largely consumed by cells in response to more UFAs. In addition, acetyl coenzyme A (Acetyl CoA) was accumulated in both strains with UFAs addition. Acetyl CoA is the key intermediate in the central metabolism of yeast, and it is used to synthesize a vast amount of functional products, however, its level is usually kept in equilibrium in normal conditions [22]. The accumulation of acetyl CoA confirmed again that yeast physiology is disturbed by exogenous UFAs. A global reduction of amino acid contents was observed in both strains, especially in the early fermentation, including phenylalanine, methionine, leucine, isoleucine, valine, lysine, asparagine, aspartate, and serine, suggesting that the most amino acids are used for cells proliferation. The intracellular contents of LA, OA, and ALA are correspondingly increased due to the incorporation of exogenous UFAs, which results in decreased production of saturated fatty acids in cells (except for tetradecanoic acid in BDX strain). This result is in agreement with the data of the contents of medium-chain fatty acids (MCFAs) in the wines (Table S7).

### 3.4. Effects of Pre-Fermentative Addition of UFAs Mixture Genes Expression Related to Aroma Compounds Formation

**Figure 4:**
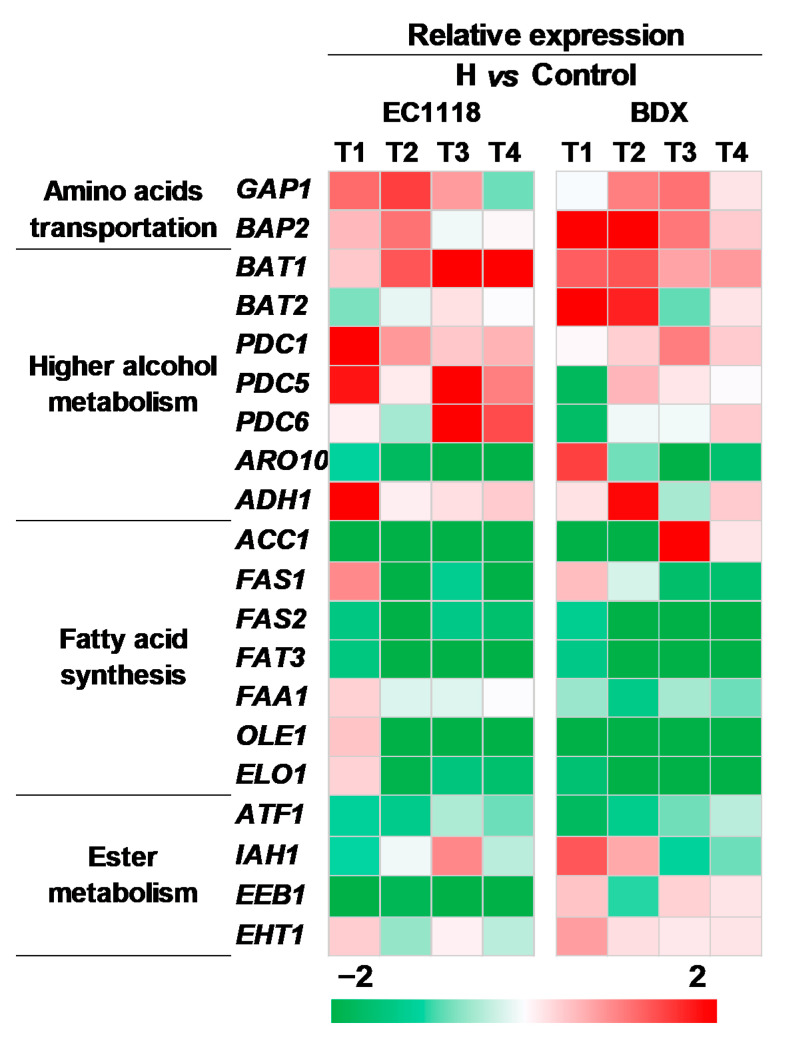
The comparison of aroma gene expressions in EC1118 and BDX strains fermented in high-UFAs addition vs. the control juice. T1, T2, T3, and T4 represented the mid-exponential phase, the early-stationary phase, the mid-stationary phase, and the end of fermentation, respectively.

To reveal the mechanism of UFAs regulating aroma at molecular level, the relative expression levels of twenty genes associated with aroma syntheses were quantified at different stages of fermentation by real-time PCR (Figure 4). These genes involved in amino acid transportation (*GAP1*, *BAP2*) and higher alcohol biosynthesis (*BAT1*, *BAT2*, *PDC1*, *PDC5*, *PDC6*, *ARO10*, and *ADH1*), fatty acid biosynthesis (*ACC1*, *FAS1*, *FAS2*, *FAT3*, *FAA1*, *OLE1*, and *ELO1*) and ester biosynthesis (*ATF1*, *IAH1*, *EEB1*, and *EHT1*). As showed in Figure 4, the expressions of all determined genes were significantly varied in the whole fermentation, and some (*BAT2*, *PDC5*, *PDC6*, *ARO10*, and *EEB1*) presented different patterns between both strains. Specifically, the genes related to amino acid transportation (*GAP1*, *BAP2*) and higher alcohol biosynthesis (*BAT1*, *PDC1*, and *ADH1*) were up regulated in both strains; while the genes of fatty acid biosynthesis (*ACC1*, *FAS1*, *FAS2*, *FAT3*, *FAA1*, *OLE1*, and *ELO1*) and acetate ester biosynthesis (*ATF1*) were down-regulated. These changes are corresponding to the varied profiles of higher alcohols, medium-chain fatty acids, and acetate esters in two strains. The noticeable strain-specific effect was observed in *EEB1*, which encoded major ethanol acyltransferase and is responsible for the biosynthesis of MCFA ethyl esters; its expression level was up-regulated in the BDX strain while down-regulated in the EC1118 strain. 

## 4. Discussion

To gain deep understanding of the mechanism of UFAs regulating chemical and aromatic compounds, we investigate the effect of UFAs addition on intra-metabolites, major fermentation products, and aromatic compounds profiles of Cabernet Sauvignon wine fermented with two *S. cerevisiae* strains (EC1118 and BDX), respectively. The results shows that pre-fermentative addition of UFAs mixture significantly alters the physiological and energetic state of the cell, and affects the metabolism of carbon and nitrogen source, which consequently modify the chemical and aromatic component of the final wines.

Consistent with previous results [23], UFAs supplementation promoted yeast growth and biomass of both strains (Figure 1), but unexpectedly, the promotion did not correspondingly increased fermentation activity. Conversely, decreased sugar consumption rate and prolonged fermentation duration time, especially in high-UFAs added fermentation, was noticed. This is inconsistent with the previous result of Varela, Pizarro, and Agosin [24] who proposed that viable yeast cell concentration is positive with the fermentation rates in wine fermentation. We ascribed this inconsistency to the changes of metabolism patterns of yeast in response to excess UFAs in the environment. Under anaerobic fermentation condition, the absence of oxygen suppresses the cells de novo synthesis of UFAs, and yeast cells have to directly uptake UFAs from grape juice in order to maintain the cell membrane integrity and resist various fermentative stresses, while the uptake of UFAs is ATP-dependent [25]. Moreover, once in the cytoplasm, the cells need to compartmentalize UFAs into the peroxisome and to use ATP again [26]. The requirement for abundant ATP would activate the flux of glycolysis pathway because it is the only way for yeast to produce ATP under anaerobic condition [25], and consequently alter the physiological and energetic state of cells. The decreased levels of glycolysis intermediates and ATP in cells support this conclusion. The activation of ATP production leads to two consequences. Firstly, intracellular NADH levels are increased and cause redox imbalance (Figure 3). Secondly, the NADH-consume reactions would be activated and the NAD^+^ dependent reactions are restrained because cells need to re-oxidize excess NADH to NAD^+^ [27]. This explains the increased production of glycerol and decreased acetic acid synthesis observed (Table 1). Glycerol is the main metabolite produced by yeasts to maintain the intracellular redox balance (NADH/NAD^+^ turnover) [28]. Under anaerobic conditions at high glucose concentration, such as wine fermentation condition, the cytosolic redox balance is mainly restored by glycerol formation [27], which consequently lead to decreased acetic acid production [29]. The link of decreasing acetic acid and increased glycerol production was observed in previous work [30]. We also observed the slight increment of ethanol content in response to UFAs addition. This finding implied that under the fermentation condition with more exogenous UFAs, glycerol and ethanol syntheses is the strategy for cells to re-oxidize excess NADH, in which, glycerol plays the major function. The large production of ATP is usually accompanied with increased cell growth, which is in agreement with the results of biomass concentration in Figure 1. Certainly, the increased resistance of cells to stress conditions, such as ethanol toxicity due to incorporating more UFAs, is possibly another reason for increased biomass. Under anaerobic condition, the metabolism of TCA cycle is limited. The decrement of TCA cycle intermediates might be drawn for producing biomass and amino acids.

The analysis of intracellular fatty acids indicated that *S. cerevisiae* cells promptly uptake added UFAs and incorporated them into cells, which consequently down-regulate functional genes expressions (*ACC1*, *FAS1*, *FAS2*, *FAT3*, *FAA1*, *OLE1*, and *ELO1*) to inhibit the biosynthesis of saturated fatty acids (SFAs), because cells don’t need de novo synthesis of UFAs from SFAs [31]. This can explain the decreased levels of MCFAs in the medium observed in this study. The disturbance of fatty acids syntheses probably leads to the accumulation of acetyl-CoA in cells because acetyl-CoA is the precursor of fatty acid synthesis. The research of Casu et al. [25] indicated that *S. cerevisiae* might have an alternative anaerobic pathway to β-oxidation, and lead to UFAs (LA) broken down into acetyl-CoA molecules under anaerobic conditions, which leads to acetyl-CoA accumulation in cells. The metabolism of amino acids is of particular interest from a winemaking perspective because they are not only cell building blocks, but also the precursors of aroma compounds [32]. We found that the contents of most amino acids in cells were less compared to the control cells in the early stage, except for glutamate, which was strengthened by UFAs addition (Figure 3). As yeast-preferred amino acids, glutamate can be synthesized from other amino acids through transamination reaction [33]. So, at the beginning of fermentation, most amino acids are consumed to form preferred amino acids for cell construction, and leads to the global reduction of amino acids (Figure 3). The increased expressions of *BAT1*, *PDC1*, *PDC5*, and *ADH1* in Ehrlich pathway confirmed that the pathway is activated by UFAs addition, which led to high productions of higher alcohols (Table 2). Another explanation for increase of higher alcohols might be that the formations of higher alcohols can re-oxidize excess NADH to NAD^+^, because the *ADH* gene used NADH as the cofactor to catalyze the last step in the Ehrlich pathway [34].

Acetate esters are synthesized from acetyl-CoA with ethanol or a higher alcohol catalyzed by alcohol acetyltransferases (encoded by *ATF1* and *ATF2*). The inhibition of *ATF1* expression by single or mixed UFAs addition has been confirmed by different authors [8,35]. Our results showed that although the both precursors (acetyl-CoA and higher alcohol) are excessive in cells, the production of acetate esters are still suppressed in UFAs added fermentation. This statement confirmed that *ATF1* is the key factor determining the synthesis of acetate esters, not precursor availability. It is worth noting that *IAH1* was up-regulated in the early stage of BDX fermentation and in the late stage of EC1118 fermentation, which might partially explain the decrement of acetate esters in UFAs added fermentation because *IAH1* encodes an esterase to hydrolyse acetate esters [36]. MCFA ethyl esters are formed from the reactions of medium-chain fatty acid with ethanol catalyzed by two acyl-CoA: ethanol *O*-acyltransferases (encoded by *EHT1* and *EEB1*), in which *EEB1* has the main function, while *EHT1* plays a minor role [1,22]. Unlike acetate esters, the formation of MCFA ethyl esters is the precursor’s (MCFAs) dependent, because increased production of precursors (MCFA) resulted in a high amount of MCFA ethyl esters, while overexpressing *EHT1* and *EEB1* did not significantly increase their formation in wine [37]. While the data presented here indicated that the availability on MCFAs is not limiting for ethyl ester production as their total concentration is above 22 fold higher than that of ethyl esters. More important, we found that the response expression of *EEB1* to UFAs is strain-specificity. In contrast to the strain of EC1118, UFAs additionally favored the expression of *EEB1* in BDX strain, which is corresponding to the high production of MCFAs ethyl esters compared to the control. This also confirmed the prior results that *EEB1* expression is a key factor determining the synthesis of MCFA ethyl esters [8,38]. The strain-specific characteristic of UFAs on MCFA ethyl esters might explain the inconsistent data of MCFA ethyl ester levels in different UFAs added wine fermentation [10,12,39]. Revealing the differences of *EEB1* sequence and regulatory mechanisms in the two strains might be helpful to elucidate the distinct mechanism of UFAs regulating MCFA ethyl esters syntheses, and this is underway in our laboratory.

## 5. Conclusions

The results obtained in this work demonstrated that the pre-fermentative addition of UFAs mixture significantly influenced the physiological and energetic state of cell due to uptake exogenous UFAs, which affected the central metabolism of *S. cerevisiae* (glycolysis pathway, TCA cycle, amino acids, and redox balance) and the expressions of most volatile syntheses genes. The responses of cells correspondingly modified the fermentation performance, chemical and volatile compounds in final wine. The strains of EC1118 and BDX produce a similar aroma profile with the exception of MCFA ethyl esters which shows the strain-specificity characteristics due to the distinct expression of *EEB1*. Our results highlight the effectiveness of modulating UFAs in regulating volatile and non-volatile syntheses, and also suggested that fine adjusting UFAs content in grape must combined with inoculating proper wine yeast is a promising strategy to modify the quality and the aromatic diversity of wine, which provides an alternative way to meet the expectations of wine consumers for various aromatic qualities.

## Figures and Tables

**Table 1:** Effects of different levels of unsaturated fatty acids (UFAs) mixtures on yeast fermentation activity and major fermentation products of EC1118 and BDX strains.

Treatments	E_Control	E_L	E_H	B_Control	B_L	B_H	*p* Value
Fermentation duration time (h)	326	326	446	326	446	446	
Maximum biomass (g DCW/L)	2.44 ± 0.09 a	2.59 ± 0.06 a	2.77 ± 0.14 b	2.36 ± 0.09 a	2.65 ± 0.04 b	2.80 ± 0.09 b	*
Maximum growth rate (g DCW/L/h)	0.028 ± 0.001 a	0.028 ± 0.001 a	0.030 ± 0.002 a	0.035 ± 0.001 b	0.036 ± 0.001 b	0.033 ± 0.002 ab	*
Maximum fermentation rate (g/L/h)	1.94 ± 0.05 a	1.96 ± 0.03 a	1.61 ± 0.03 b	1.93 ± 0.10 a	1.86 ± 0.06 a	1.61 ± 0.05 b	*
Glycerol (g/L)	9.32 ± 0.3 a	10.23 ± 0.2 b	10.57 ± 0.18 c	8.78 ± 0.27 a	10.26 ± 0.06 b	10.45 ± 0.21 c	*
Ethanol (% *v*/*v*)	13.94 ± 0.25 a	14.38 ± 0.05 b	14.57 ± 0.05 b	13.86 ± 0.46 a	14.49 ± 0.08 b	14.72 ± 0.19 b	*
Citric acid (g/L)	0.22 ± 0.01 ab	0.21 ± 0.01 a	0.21 ± 0.01 ab	0.21 ± 0.01 a	0.21 ± 0.02 a	0.21 ± 0.01 a	*
Malic acid (g/L)	3.10 ± 0.06 a	3.28 ± 0.22 a	3.52 ± 0.03 b	3.58 ± 0.22 b	3.14 ± 0.07 a	3.39 ± 0.29 a	*
Succinic acid (g/L)	3.60 ± 0.04 a	3.79 ± 0.22 ab	3.87 ± 0.01 ab	4.39 ± 0.23 c	3.93 ± 0.05 b	4.48 ± 0.20 c	*
Acetic acid (g/L)	0.51 ± 0.07 a	0.42 ± 0.02 b	0.43 ± 0.01 b	0.39 ± 0.03 b	0.31 ± 0.01 c	0.24 ± 0.04 d	*

Values (mean ± SD, *n* = 3) of the same compound followed with the same lowercase letters indicate no significant difference (*p* < 0.05, Duncan’s test). Abbreviations: E_Control, E_L, and E_H represented the control, low-UFAs, and high-UFAs added wines fermented by EC1118 strain, respectively; B_Control, B_L, and B_H represented the control, low-UFAs, and high-UFAs added wines fermented by BDX strain, respectively. * means the least significant difference < 0.05.

**Table 2:** Effects of different levels of UFAs mixtures on volatile compounds (OAV > 1) and total concentrations of higher alcohol, esters, and medium-chain fatty acids in wine after alcoholic fermentation.

Compounds	E_Control	E_L	E_H	B_Control	B_L	B_H	*p* Value	Thresholds
Isobutyl alcohol (mg/L)	31.87 ± 2.92 a	47.29 ± 6.01 b	72.35 ± 9.53 c	43.62 ± 7.23 b	50.251 ± 7.48 b	106.57 ± 11.05 d	*	40
Isoamyl alcohol (mg/L)	295.05 ± 28.92 a	411.09 ± 45.14 bc	524.72 ± 65.58 d	355.38 ± 13.66 b	448.63 ± 56.93 c	645.46 ± 72.21 e	*	60
2-Phenylethanol (mg/L)	62.26 ± 8.29 a	72.11 ± 8.59 b	75.47 ± 11.07 b	91.04 ± 9.29 c	87.55 ± 8.66 c	106.83 ± 5.67 d	*	10
Total higher alcohols (mg/L)	369.04 ± 61.55 a	511.30 ± 48.09 b	680.04 ± 82.44 c	428.89 ± 63.66 a	587.17 ± 71.45 b	838.11 ± 117.40 d	*	
Total C6 alcohols (mg/L)	0.49 ± 0.01 ab	0.54 ± 0.01 ab	0.62 ± 0.18 bc	0.47 ± 0.03 a	0.54 ± 0.01 ab	0.74 ± 0.21 c	*	
Ethyl acetate (mg/L)	48.84 ± 3.83 a	37.40 ± 2.85 b	34.87 ± 4.53 b	67.40 ± 9.09 c	37.68 ± 3.69 b	34.21 ± 2.56 b	*	7.50
Isoamyl acetate (mg/L)	1.79 ± 0.24 a	0.88 ± 0.09 b	0.65 ± 0.101 c	3.51 ± 0.17 d	0.89 ± 0.13 b	1.00 ± 0.09 b	*	0.16
Phenethyl acetate (mg/L)	0.59 ± 0.08 a	0.31 ± 0.04 b	0.23 ± 0.03 c	0.76 ± 0.13 d	0.35 ± 0.03 b	0.37 ± 0.05 b	*	0.25
Total acetate esters (mg/L)	51.29 ± 3.98 a	38.64 ± 2.95 b	35.81 ± 4.63 b	73.29 ± 10.90 c	38.97 ± 3.84 b	35.53 ± 2.84 b	*	
Hexanoic acid (mg/L)	47.78 ± 5.83 a	29.70 ± 3.08 b	34.981 ± 4.42 c	41.32 ± 0.85 d	26.98 ± 2.45 be	24.45 ± 2.22 e	*	0.42
Octanoic acid (mg/L)	98.59 ± 3.78 a	58.06 ± 6.93 b	42.565 ± 4.97 c	73.63 ± 4.03 d	43.30 ± 5.26 c	22.13 ± 3.26 e	*	0.5
Decanoic acid (mg/L)	77.23 ± 3.60 a	48.48 ± 5.62 b	30.48 ± 4.27 c	42.43 ± 4.47 d	25.34 ± 3.59 e	11.79 ± 0.85 f	*	1
Isobutyric acid (mg/L)	4.35 ± 0.68 a	5.04 ± 0.75 b	5.07 ± 0.46 b	3.76 ± 0.44 c	4.88 ± 0.55 ab	4.93 ± 0.49 ab	*	3
Total medium-chain fatty acids (mg/L)	224.91 ± 3.88 a	140.44 ± 15.37 b	106.24 ± 14.69 c	165.37 ± 5.38 d	100.50 ± 11.09 e	58.13 ± 3.43 c	*	
Ethyl hexanoate (μg/L)	335.98 ± 31.38 a	219.4 ± 22.01 b	162.94 ± 20.31 c	528.81 ± 67.73 d	205.82 ± 27.75 b	211.26 ± 28.85 b	*	80
Ethyl octanoate (μg/L)	619.74 ± 74.42 a	397.47 ± 53.15 b	351.04 ± 55.95 b	395.2 ± 93.44 b	408.8 ± 52.02 b	380.99 ± 53.86 b	*	2
Ethyl decanoate (mg/L)	1.93 ± 0.22 a	1.49 ± 0.21 bc	1.56 ± 0.21 b	1.02 ± 0.08 d	1.33 ± 0.15 c	1.53 ± 0.18 b	*	0.2
Total ethyl esters (mg/L)	3.49 ± 0.27 a	2.45 ± 0.30 bc	2.69 ± 0.24 b	1.92 ± 0.11 d	2.28 ± 0.35 c	2.53 ± 0.29 bc	*	
Methyl octanoate (mg/L)	6.16 ± 0.83 a	5.68 ± 0.60 b	3.61 ± 0.46 cd	3.32 ± 0.35 c	3.54 ± 0.19 cd	3.92 ± 0.47 d	*	0.2
β-Damascenone (μg/L)	218.57 ± 12.02 ab	231.98 ± 25.61 abc	205.01 ± 19.47 a	259.31 ± 12.93 c	247.94 ± 21.44 c	238.19 ± 35.1 bc	*	0.14
3-(Methylthio)-1-propanol (mg/L)	1.14 ± 0.14 a	1.72 ± 0.12 b	1.73 ± 0.22 b	2.96 ± 0.38 c	1.75 ± 0.24 b	3.68 ± 0.24 d	*	1

Values (mean ± SD, *n* = 3) of the same compound followed with the same lowercase letters indicate no significant difference (*p* < 0.05, Duncan’s test). Abbreviations: E_Control, E_L, and E_H represented the control, low-UFAs, and high-UFAs added wines fermented by EC1118 strain, respectively; B_Control, B_L, and B_H represented the control, low-UFAs, and high-UFAs added wines fermented by BDX strain, respectively. * means the least significant difference < 0.05.

## Data Availability

Not applicable.

## Supplementary Materials

The following are available online at https://www.mdpi.com/2304-8158/10/2/277/s1, Table S1: Calibration curves for quantification of glycerol, ethanol, acetic acid, citric acid, succinic acid, and malic acid in this study, Table S2: Quantitative and quantified information of fatty acids in this study, Table S3: Quantitative ion, quantitative standards, and calibration curves for quantification of volatile compounds in this study, Table S4: Quantitative and quantified information of yeast intracellular amino acids in this study, Table S5: Quantitative and quantified information of yeast intracellular compounds involved in glycolysis, tricarboxylic acid cycle, and energy metabolism in this study, Table S6: Description of genes detected in this work, Table S7: Effect of different levels of UFAs on volatile compounds in wine after alcoholic fermentation.
